# The Danaid Theory of Aging

**DOI:** 10.3389/fcell.2021.671208

**Published:** 2022-01-03

**Authors:** Maarten J. Wensink, Alan A. Cohen

**Affiliations:** ^1^ Interdisciplinary Center on Population Dynamics, University of Southern Denmark, Odense, Denmark; ^2^ Department of Family Medicine, Research Centre on Aging, CHUS Research Centre, University of Sherbrooke, Sherbrooke, QC, Canada

**Keywords:** evolution, aging, development, theory, constraint, maintenance, senescence

## Abstract

The classical evolutionary theories of aging suggest that aging evolves due to insufficient selective pressure against it. In these theories, declining selection pressure with age leads to aging through genes or resource allocations, implying that aging could potentially be stalled were genes, resource allocation, or selection pressure somewhat different. While these classical evolutionary theories are undeniably part of a description of the evolution of aging, they do not explain the diversity of aging patterns, and they do not constitute the only possible evolutionary explanation. Without denying selection pressure a role in the evolution of aging, we argue that the origin and diversity of aging should also be sought in the nature and evolution of organisms that are, from their very physiological make up, unmaintainable. Drawing on advances in developmental biology, genetics, biochemistry, and complex systems theory since the classical theories emerged, we propose a fresh evolutionary-mechanistic theory of aging, the Danaid theory. We argue that, in complex forms of life like humans, various restrictions on maintenance and repair may be inherent, and we show how such restrictions are laid out during development. We further argue that there is systematic variation in these constraints across taxa, and that this is a crucial factor determining variation in aging and lifespan across the tree of life. Accordingly, the core challenge for the field going forward is to map and understand the mosaic of constraints, trade-offs, chance events, and selective pressures that shape aging in diverse ways across diverse taxa.

## Introduction

An evolutionary theory of aging should answer two key questions. First, why could aging evolve, given that, all else being equal, an individual’s fitness should be maximized by living as long as possible? Second, why do patterns of aging vary across the tree of life the way they do (Omotoso et al., 2021)? The classical evolutionary theories of aging have long provided a convincing answer to the first question (Medawar, 1952; Williams, 1957; Hamilton, 1966; Kirkwood, 1977). However, as we learn more about the diversity of aging patterns across the tree of life and the diversity of mechanisms, it is increasingly clear that the classical theories do not provide a sufficient answer to the second question. Additionally, other answers to the first question are possible. Here, we propose a novel theory, The Danaid Theory of aging, that builds on existing theory, links mechanisms with evolution, and can simultaneously answer both questions. It integrates the previous theories with a modern understanding of development, aging biology, complex systems, and genetic control, contextualizing when previous theories may be key drivers, and when other forces may dominate the evolution of aging and lifespan.

The Danaid theory suggests that there are taxon-specific constraints on the ability of organisms to maintain themselves indefinitely, often arising from the inherently complex systems nature of organisms. This is complementary to a declining force of natural selection with age, but does not depend on it. Accordingly, we propose a framework in which specific types of mechanistic constraints complement the declining force of selection to explain the diversity of aging patterns, showing that evolutionary and mechanistic theories are inextricably intertwined. We start by establishing some preliminaries—a taxonomy of aging theories, definitions of aging, etc. Readers who wish to dive straight into the subject matter may wish to proceed directly to *Systemic Constraints on Physiology and Evolution*. We then address the necessary elements of evolutionary theory, followed by a consideration of how systemic constraints could influence aging, and a discussion of how such constraints may emerge from the complex nature of life. Finally, we consider how this theory interacts with our knowledge about the diversity of aging processes across taxa.

## Preliminaries

### A Taxonomy of Aging Theories

**FIGURE 1:**
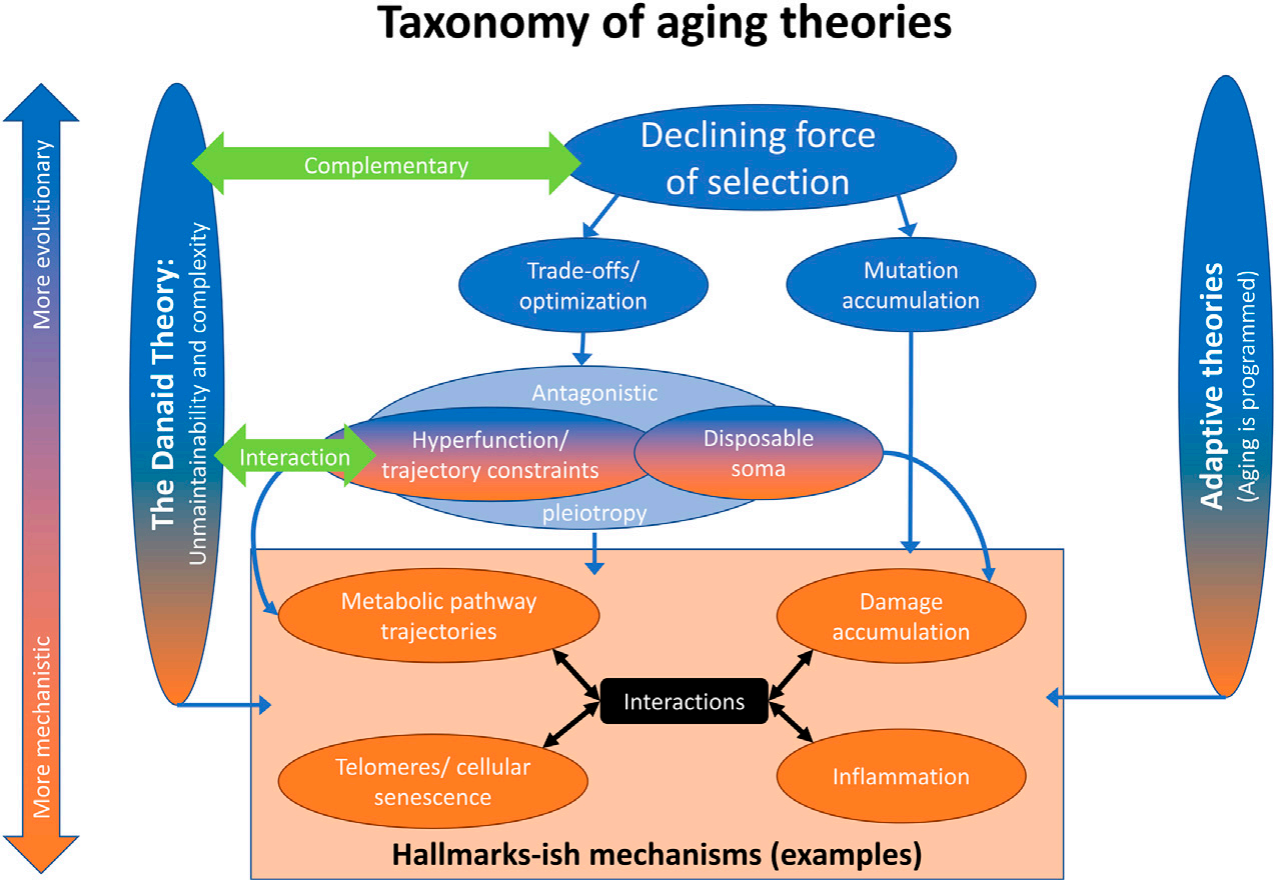
A taxonomy of aging theories. Aging can be viewed from a more mechanistic or more evolutionary angle (vertical direction). Most approaches consider elements of both. Aging can also be viewed from more adaptive or less adaptive angles (horizontal direction). The programmed theories ascribe a direct function to aging; they are the most adaptive. The current theoretical framework, i.e. the various theories in the middle, consider aging a phenomenon that follows from evolutionary pressures, but is not as such selected for. The Danaid theory rather sees aging in part as the result of the physiological layout of organisms, with only limited malleability through selection.

While the classical theories of aging are often listed as mutation accumulation (Medawar, 1952), antagonistic pleiotropy (Williams, 1957), and the disposable soma theory (Kirkwood, 1977) (in large part to pay hommage to three landmark papers in the field), we believe there is now a clear consensus for a new way to think about these classical theories, as shown in Figure 1. This taxonomy of theories is not meant to be exhaustive, but does recognize other, more recent theoretical advances. On the one hand, there are the programmed/adaptive theories of aging (Goldsmith, 2012; Libertini, 2015; Mitteldorf, 2018). Though they remain popular with molecular biologists and some physicists, they have been debunked by evolutionary biologists, including ourselves and others (Austad, 2004; Cohen, 2015; Kowald and Kirkwood, 2016). We do not consider them futher.

The classical theories of aging all stem from a single principle, the declining force of selection with age (“selection shadow”): because future events cannot affect past reproduction, as organisms reproduce selection lessens with progressing age (Hamilton, 1966; Wensink et al., 2017a). Within this broad principle, there are trade-off/optimality theories (Stearns, 1989; Parker and Smith, 1990; Partridge and Barton, 1993) and mutation accumulation (Medawar, 1952). Trade-off/optimality theories hold that aging is a byproduct of maximizing fitness, generally through trade-offs between fertility/reproduction/condition early in life and the ability of the organism to maintain itself indefinitely. Mutation accumulation does not invoke an advantage linked to aging, but quite simply posits that weak selection against late-acting deleterious mutations increases the load of mutations with late-life-specific effects.

Within optimality theories, antagonistic pleiotropy (Williams, 1957) posits a genetic mechanism whereby a single allele might have constrasting effects on fitness early versus late in life. This is a special case; the broader principles of optimality/trade-offs can be expressed through multiple genes with contrasting effects, for example (Parker and Smith, 1990; Partridge and Barton, 1993). The disposable soma theory (Kirkwood, 1977) is another special case of trade-offs/optimality, wherein the major mechanistic manifestation is through trade-offs in resource allocation. The hyperfunction theory (Blagosklonny, 2012; Maklakov and Chapman, 2019) classically posits that processes that start earlier in life continue with, or set the organism on a trajectory to, aging further down the road. In this type of trade-off, excessive late function is a price paid for appropriate early development, and alternative trajectories would show slower aging with lower early function. Slightly more broadly, we consider the hyperfunction theory a special case of a general principle: biological processes over the course of an organism’s lifespan are generally hard to time precisely, particularly after development, and thus result more from trajectories than from precise temporal optimization (Cohen, 2004; Kirkwood and Melov, 2011; Wensink, 2013), much as artillery gunners can adjust the direction and angle of a cannon, but lack control over the cannonball after it has been fired.

Antagonistic pleiotropy as depicted in Figure 1 largely but not completely covers both the disposable soma and hyperfunction theories. The lack of complete coverage acknowledges the potential for non-genetic mechanisms, or for mechanisms that are related to genetics in substantially more complex ways than typically considered under antagonistic pleiotropy (effects that are high-dimensionally epistatic as well as contingent on environment). The adaptive hitchhike hypothesis posits that slow aging is a byproduct of other adaptations (Omotoso et al., 2021), and thus is also consistent with optimality approaches. All of these theories, as well as our Danaid theory and adaptive theories, can then be related to purely mechanistic theories of aging, such as those contained in the Hallmarks framework (López-Otín et al., 2013). For example, it has been proposed that the apparently programmed nature of cellular senescence supports adaptive explanations for aging (Milewski, 2010). While we disagree with this contention, the nature of the mechanisms of aging can inform our evaluation of the various evolutionary theories.

### Proximate Versus Ultimate Theories of Aging?

Descriptions of the mechanisms of aging are usually considered proximate explanations, as opposed to ultimate evolutionary explanations (Gems and Partridge, 2013). An evolutionary explanation would give the “what” and “why”; the mechanisms would provide the details of the “how”. However, evolutionary models of aging consistently show that essentially any outcome can occur depending on the proximate mechanisms that constrain the range of possible evolutionary outcomes (Baudisch, 2008; Wensink et al., 2014a; Wensink et al., 2014b): depending on constraints, evolution can produce senescence, no senescence, or negative senescence, as well as variation within these categories. While studying mechanisms without evolution indeed means studying proximate but not ultimate explanations of aging, the reverse is not true: studying evolution without mechanisms does not yield an ultimate explanation, but rather no explanation at all. Although “nothing in biology makes sense except in the light of evolution” (Dobzhansky, 1973), nothing in evolution makes sense without the mechanisms. An ultimate theory of aging, if it exists, is found at the intersection of evolutionary and mechanistic forces. Any convincing evolutionary theory of aging must incorporate not only what is known about comparative demography and natural selection, but also what is known about mechanisms and their distribution across taxa.

Most scientists appear to agree with the above, and see existing evolutionary theories of aging as “solving the paradox”: how could aging evolve given its apparently detrimental effect on fitness? The classical theories of aging do provide a plausible solution to the paradox. Yet as soon as we want to do more than solving the paradox, the mechanisms start to matter.

Even those who attempted to just solve the paradox seemed to feel that mechanisms mattered. Hamilton (Hamilton, 1966) sought to explain senescence through calculating the effect on Darwinian fitness of a change in mortality or fecundity at an isolated age. From the decline in the magnitudes of these sensitivities with age Hamilton inferred that senescence is inevitable. Yet he also gave a mechanistic justification:

“Consider four hypothetical genes in man, (…) age-limited in the following way: each gives complete immunity against some lethal disease but only for one particular year of life. Suppose the first gives immunity for the first year, the second for the fifteenth, the third for the thirtieth, and the fourth for the forty-fifth. What are the relative selective advantages of these genes?”

There is no description of how this would work mechanistically, just “a gene”. While we appreciate that Hamilton merely wished to justify the presentation of a set of mathematical results, later developments force us to take a broader perspective (Kirkwood, 1977; Noble, 2013; Wensink, 2013; Noble et al., 2014), focusing on the way an organism is built and how it functions, which depends on more than DNA alone. No gene gives immunity for one specific year of life.

The disposable soma theory, taking a thermodynamics perspective, comes closer to actual mechanisms. Still, nowadays its pure focus on trade-offs, in particular resource trade-offs, seems too narrow in scope (Cohen et al., 2019), in particular given that the co-existence of multiple trade-offs can change the outcome expected under individual trade-offs (Cohen et al., 2017), further discussed in subsection *Allocation Theory*.

In short, develomental biology and the role of DNA, for example, are seen in a much different light now compared to several decades ago. In this paper we continue the search for a theory of the evolution of aging more firmly rooted in the mechanisms of organismal physiology, informed where possible by accepted principles from other disciplines, e.g. physics and chemistry, in the tradition of D’Arcy Wentworth Thompson’s *On Growth and Form* (Thompson, 2014). In particular, we suggest that multiple complex constraints evolve for reasons largely unrelated to lifespan, but nonetheless shape the relative “maintainability” of various taxa, and thereby their lifespans (Table 1).

**TABLE 1:** A comparison of evolutionary(-mechanistic) aging theories.

	Adaptive theories	Classical theories	The danaid theory
**Role of natural selection**	Aging is selected for	Aging is a byproduct of declining selection with age	Aging is inevitable, at least in some taxa, though selection could change its rate
**Role of constraints**	Constraints? What constraints?	Of course there are constraints, now let’s talk about something interesting	Constraints vary across taxa and are key to understanding the interplay between mechanisms and selection
**Integration of mechanisms**	Mechanisms suggest adaptation	Mechanisms are generally incidental, but many support trade-offs	The evolution of aging can’t really be understood without considering mechanisms and their variation across taxa
**Role of trade-offs**	Might affect how much aging is adaptive?	Core to DS and AP theories	Importance is highly variable across taxa
**Relationships to other theories**	Rejects other theories	Depends on which one, but generally consider themselves sufficient together	Incorporates classical theories as a partial but not global explanation
**Explanation of taxonomic diversity in aging**	Not on the radar	Not considered beyond basic trade-offs and life-history continua	Considers explanation of taxonomic diversity a core task of an aging theory
**Role of development**	Both development and aging are programmed and can be fine-tuned independently by selection; not therefore necessary to consider development to understand aging	Crucial for the hyperfunction theory; early-late trade-offs also consistent with AP	Considers taxon-specific developmental programs as potential key constraints on how aging can evolve
**Aging as damage**	Damage is a byproduct of programs to age	Damage is often considered crucial, but is not an inevitable conclusion of the theories	Damage is insufficient to understand aging. Unclear whether damage is cause, consequence, or both; this may vary across taxa
**Role for complex systems**	Not much	Not much	The nature of organisms as complex systems is a key contributor to taxon-specific constraints
**Predictions**	Support generally comes from simulations rather than empirical research; testable predictions needed	Substantial support for predictions in certain examples, but the universality of the explanations is questionable	Few broad generalizations expected, so specific predictions are hard. Empirical patterns consistent or inconsistent with the theory, such as concordance between patterns of aging and selection pressure, will nonetheless provide tests

Notes: MA: mutation accumulation; AP: antagonistic pleiotropy; DS: disposable soma

### What Is Aging?

**FIGURE 2:**
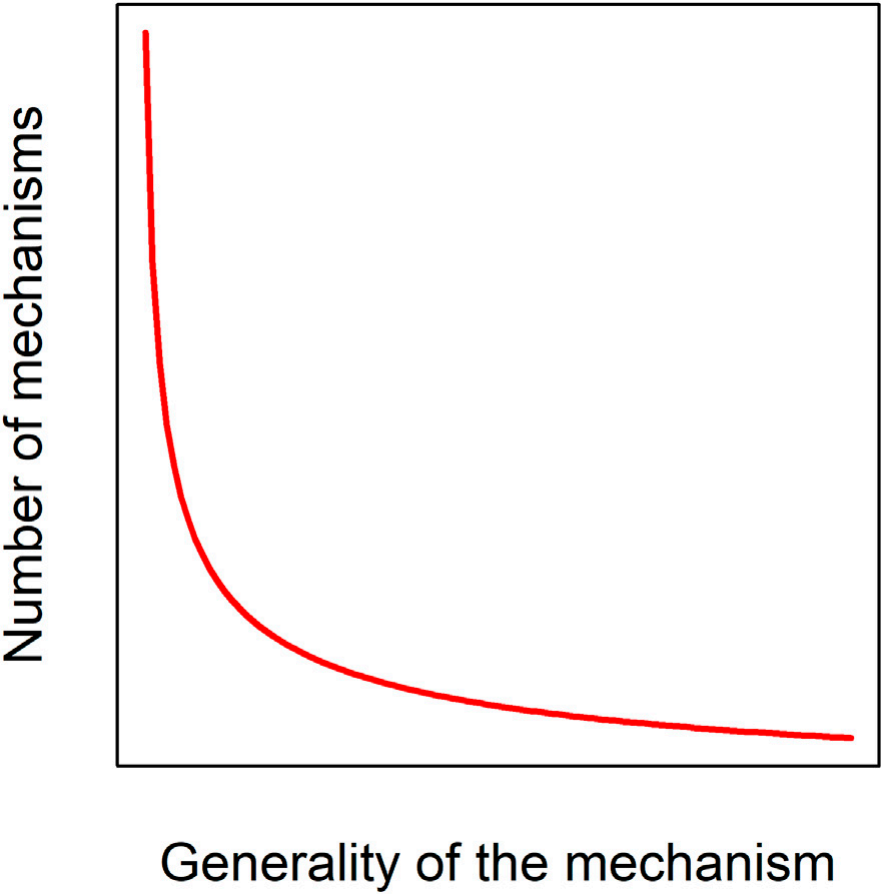
Aging mechanisms likely follow a power-law distribution. Only a small number of mechanisms or pathways, such as DNA damage, are likely to have large, consistent effects across many species, individuals, and environments; a much larger number could have effects that are specific to a given taxon, genetic background, or environmental background.

Despite our clear intuition for what aging is, there are major disagreements among researchers as to its nature and definition (Cohen et al., 2020a), and there are important arguments against the idea of aging as a unified biological phenomenon (Cohen et al., 2020b). A demographic definition such as “monotonic increases in age-specific mortality” includes cases where the mechanisms behind the demographic patterns have little to do with traditional concepts of aging biology. For example, fish and tree mortality are size dependent, a phenomenon largely unrelated to traditional aging mechanisms like declines in tissue function. Additionally, it is hard to know from demographic data what mechanistic aging might look like were we able to keep enough individuals alive longer in a protected environment. A more mechanistic definition, such as “age-related declines in organismal function due to the hallmarks of aging” [with the hallmarks described as in (López-Otín et al., 2013)], runs the risk of missing mechanisms that have yet to be discovered, or that play a role in other taxa, since the hallmarks were tailored to mammals. Indeed, there is every reason to expect a diversity of aging mechanisms across taxa, across individuals, and across environments (Cohen, 2018), presumably following a power-law distribution (Figure 2). An evolutionary definition such as “declines in age-specific fitness” might seem appropriate in this context, but poses the challenge of being hard to measure in many contexts, and hard to relate to mechanisms. We here focus on aging as a progressive and intrinsic decline in physiological function, i.e. an organism’s ability to maintain homeostasis and respond to its environment. We nonetheless integrate information from different sources, including demographic, and do our best to acknowledge that these shifting data types may not always reflect the same underlying phenomenon of aging (Cohen et al., 2020b).

A popular idea is that aging is caused by the accumulation of somatic damage with age (Kirkwood and Austad, 2000; López-Otín et al., 2013). While appealing, this notion requires a clear definition of damage, which is problematic. López-Otín et al. (López-Otín et al., 2013) list nine “hallmarks of aging”: genomic instability, telomere attrition, epigenetic alterations, loss of proteostasis, deregulated nutrient sensing, mitochondrial dysfunction, cellular senescence, stem cell exhaustion, and altered intercellular communication. Hallmarks like epigenetic alterations and telomere attrition consist of clear, spatial aberrations we would associate with the traditional meaning of damage. But other hallmarks, such as altered intercellular communication, deregulated nutrient sensing and mitochondrial dysfunction, have a less clear-cut spatial representation. What if a perfectly undamaged protein is in the wrong place? What if nutrient sensing is disturbed due to the expression of the wrong gene? If all aberrations such as these are classified as damage, then everything deleterious is damage and the definition becomes tautological.

Because many theories of aging do refer to damage accumulation in some way—for example, energy investment to damage repair in the disposable soma theory (Kirkwood, 1977; Kirkwood and Austad, 2000)—a definition of damage is important. The statement “aging is caused by cumulative damage” is meaningful only when adhering to a limited, three-dimensional definition of damage, which allows for alternative and/or complementary hypotheses. Malfunction may or may not have its origin in three-dimensional damage, and there is some evidence that the accumulation of damage with age may be correlation without causation (Doonan et al., 2008; Gems and Doonan, 2009). Alternatives include hyperfunction, such as unchecked growth through hormonal pathways (Blagosklonny, 2009; Blagosklonny, 2012), or other malfunctioning that does not reduce to damage in its traditional sense. For these reasons, we define damage here as a structural, physical, three-dimensional change: This definition would include DNA mutations, protein misfolding, wing damage, and tooth wear, but would exclude more general information loss, depletion of reserves, communication or regulatory errors, etc. There is increasing recognition of non-damage-based mechanisms (Gems and de Magalhães, 2021), and some are described below.

### The Germ-Soma Distinction and Asymmetric Division

A long-recognized element in aging theory is the germ-soma distinction (Weismann et al., 1891; Pen and Flatt, 2021). A division of labor exists between the cells of multicellular organisms, such that the germ cells have the task and capability to form future generations, while the somatic cells form the body of one organism, in one generation alone.

A germ-soma distinction would appear a necessary but not a sufficient condition for aging to occur. If all cells aged, life would stop, so a minimum of one cell should be precluded from aging, to serve as the basis for a next generation: the germ cell. The somatic part of the body may age, which is entirely compatible with the continuation of life, but not necessarily.

Indeed, Turke (Turke, 2013) argues that a germ-soma distinction need not be *between cells*. In unicellular organisms, there can be *regions* that are insulated from adversities, which serve as the germ regions for next generations, while other regions take a soma role, and are pared away through asymmetrical cell division. The capability of building a perfectly healthy organism should be maintained somewhere, whether or not that be in a separate cell. This manifests particularly in the asymmetric division of some single-celled organisms such as *Escherichia coli* (Jouvet et al., 2018) and yeast (*Saccharomyces cerevisiae*) (He et al., 2018).

Contrary to what might be predicted, however, not only do some organisms with germ-soma distinctions show no apparent aging (Congdon et al., 2003; Sauer et al., 2021), but some organisms without the distinction do age, at least demographically (many plants, for example) (Jones et al., 2014), which might be due to factors other than cellular biology and physiology (size, length of time a plant has its roots in the same soil). This remains speculation however; all we can say for the moment is that the germ-soma distinction does not seem to be a decisive predictor of aging at the comparative level, at least based on demographic data.

### Allocation Theory

Allocation theory is one popular framework for evolutionary thinking on aging (Kirkwood, 1977; Kirkwood and Austad, 2000; Baudisch, 2008). Like any household, company or country, organisms allocate scarce resources to competing functions, such as growth, somatic maintenance and reproduction. Resources invested in one function cannot be invested in another. There is thus expected to be strong selection to optimize resource allocation and the extent to which maintenance would be required if aging was to be halted (Wensink et al., 2012). Allocation theory has sometimes been framed in the light of a germ-soma distinction, since such a distinction presents the problem of the allocation of resource to germ (reproduction) versus soma (survival) (Kirkwood, 1977; Kirkwood and Holliday, 1979; Kirkwood and Rose, 1991).

There is little doubt that life has trade-offs to solve (Stearns, 1989), yet there are several potential problems in attempting to explain all aging phenomena in this framework. First, if there are other systemic constraints, for instance on damage detection, then it is not directly obvious how these constraints could be framed in terms of an allocation problem. Some might suggest that then resources should be allocated to damage detection, which keeps the argument within the realm of trade-offs. However, the problem cannot be reduced to this level of simplicity (see also *Systemic Constraints on Physiology and Evolution* and *Unmaintainability as an Emergent Property of the Complexity of Life*). Failing to detect damage (Hoogstraten et al., 2008) means that no allocation to repair could be made, let alone be optimized.

Second, even if aging did depend on resource allocation, there is a risk of missing the point: what drives the costs and benefits in the allocation model? Suppose that repair is simply impossible through other constraints. This is easily expressed in a resource allocation model: the gain of allocating resources to repair is zero, and the model will predict that no resources are spent on repair. But this is neither surprising nor interesting. What is interesting is the set of constraints driving the model. This principle would apply under a wide range of scenarios in which the constraint on optimal allocation is substantially larger than the range in which the allocation trade-off operates.

Both these points have been realized and subsumed in a broader field of optimality theory (Parker and Smith, 1990; Partridge and Barton, 1993). Scientists versed in optimality theory recognize that there are broader constraints to solve, and are well aware that model assumptions drive model outcomes. Yet we are wary of optimality theory becoming a *posthoc* justification, where constraints have to be assumed to create a certain model behavior or biological observation, rather than the other way round. Although few scientists would disagree that different model assumptions lead to different outcomes, this recognition is rarely sufficiently incorporated into the use of models. Since different model assumptions lead to different outcomes as diverse as senescence and negative senescence, then what are the predictions of a broader theory?

Furthermore, allocation theory is rooted in (classical) equilibrium thermodynamics: it is typically stated that entropy tends to increase in closed systems, but that organisms are open systems that take resources from their environment to oppose the entropy increase. This is true, but the newer field of non-equilibrium thermodynamics, which is about the organization of complex systems under a gradient (Schneider and Kay, 1994), states that non-equilibrium systems auto-organize so as to resist gradients as well as possible, which means that they reduce the gradient as much as possible at the local level. Within a specific range of gradient, but not outside, complex systems spontaneously assume the organization that best dissipates the gradient (Kondepudi et al., 2015). As energy flows such that gradients dissipate, then how allocable are resources? And how is this allocation achieved? The finding that ∼25% of available energy is dissipated largely unused (Brand, 2000) suggests that the practical problem of keeping biochemical gradients within a certain range may sometimes be more important than finding an appropriate allocation of the available energy. It would be recommendable for the future of allocation theory to investigate the extent to which resources can actually be allocated under specific, targeted physiological control.

Fourth, there is increasing reason to doubt the importance of trade-offs in shaping interspecific evolutionary patterns, particularly in lifespan and aging (Cohen et al., 2019; Maklakov and Chapman, 2019). Trade-offs act not only on a single resource (energy), but on multiple resources simultaneously. When multiple resources trade-off simultaneously, the force of the trade-off tends to weaken and the outcome of selection becomes less predictable (Cohen et al., 2017). This is because the efficiency of different resources for different tasks is expected to vary, opening up optimization possibilities that diminish the force of the trade-off. Also, by arguing that trade-offs have near-universal power to shape life histories, classical theory implicitly or explicitly supposes linear shapes to trade-offs. As an example, the classic van Noordwijk and de Jong paper shows a figure with linear trade-offs, and uses covariances (which are most appropriate for linear relationships) (van Noordwijk and de Jong, 1986). Such linearity may rarely be the case, with the implication that trade-offs may only operate strongly in limited regions of trait space, i.e., where the slope is intermediate and both traits can thus be optimized at a reasonable cost to the other (Bourg et al., 2019). Empirical research also shows that predicted trade-offs do not always manifest under experimental conditions (Rose, 1984).

Trade-off theory seems to predict a canalized set of pathways along which selection can move a species. It need not be perfectly canalized, but in a complex organism where genetic changes can affect multiple phenotypes with high-dimensional pleiotropy and epistasis, coherent evolution without canalization would be unmanageable, and certain regulatory networks suggest this kind of canalization (Csete and Doyle, 2004; Cohen et al., 2012). While such canalized trade-offs appear to exist in some contexts (Dantzer and Swanson, 2012), there are also clear counter-examples. For example, short lifespan has evolved repeatedly in wild killifish not through a limited set of pathways, but apparently from relaxed selection on housekeeping genes leading to a diverse array of fast-aging phenotypes (Cui et al., 2019). That is, under strong selection to develop and reproduce quickly, and with little selection for longevity, short lifespan appears to evolve differently in different lineages, with each one losing a unique set of housekeeping genes due to mutation, i.e., consistent with mutation accumulation rather than trade-offs/optimality (Figure 1). In this case, short lifespan is not due to a cost of reproduction, but a simple failure to maintain selection for maintenance.

Lastly, many trade-offs force higher-level constraints, such as when decreasing one cause of mortality increases another, leading to a constraint on mortality more globally (Pavard et al., 2019). Generally, various potential causes of death exist and, depending on their age patterns, one or the other wins out (Wensink, 2016; Wensink et al., 2017b). Causes of death are themselves the result of interactions between component causes that may be correlated (Wensink et al., 2014c), and statistical modelling suggests these interactions give rise to the typical Gompertz mortality pattern (Levy and Levin, 2014).

While there can be no doubt that trade-offs exist and may sometimes influence life histories, the ensemble of factors discussed imply that resource allocation trade-offs are not by themselves a sufficiently powerful or general explanation for the evolution of aging, and must be integrated with numerous other principles or forces (Cohen et al., 2019; Maklakov and Chapman, 2019).

### Development, Differentiation, and Totipotency

A promising mechanistic line of thought in aging—that aging is due to an extension of development, and to constraints arising from developmental processes and cell differentiation (Magalhães, 2012)—may also have relevance for the evolution of aging. Building on the idea of the germ-soma distinction, it has been suggested that the very developmental process in multicellular organisms, during which cells differentiate away from totipotency, is sufficient to explain why aging occurs, as this limits repair (Turke, 2013). This idea is supported by the observation that induced neurons generated directly from human fibroblasts have an ‘old’ epigenetic and metabolic profile, whereas induced neurons from fibroblast-derived induced pluripotent stem cells are “young” (Mertens et al., 2015). Differentiation does seem to matter, and passing through a “pluspotent” (“more potent”) state seems to allow for rejuvenation.

However, this seems more of a mechanistic problem than an evolutionary-conceptual one: there could be mechanisms to keep totipotent cells scattered across an organism which evolution simply has not yet happened on. If there are clear evolutionary benefits to totipotency, then why would an organism not retain a few totipotent cells to be able to form perfectly healthy new tissue? There may well be evolutionary advantages of such a solution, as a full-grown adult has much less to fear than immature offspring.

Another objection is that totipotency is not necessary for repair or replacement. As long as cells capable of forming a kidney are preserved, the kidney can be regenerated. As long as cells capable of forming the heart are preserved, the heart can be regenerated. It is not required that one cell can make both a heart and a kidney. Pluripotency suffices, and again the question is whether such is mechanistically achievable. While we agree with the hunch that complexity and differentiation away from totipotency play some role in aging, we argue below that it is not the loss of totipotent cells that limits the ability to regenerate and repair, but constraints at the higher level of the blueprint of an organism as a whole.

### The Nature of Evolutionary Explanations

Evolution by natural selection consists of 1) variation in 2) heritable traits that 3) affect propagation (survival and reproduction). Variation in a trait may be quantitative (e.g. size of a tail) or qualitative (e.g. red versus white tail). When organisms are observed to have some trait A rather than an alternative trait B, there are three potential explanations for this observation. First, variation could have existed but evolved away; some organisms had trait A, while others had trait B, but trait A was better adapted than B, the frequency of trait A increased at a cost to B, and now only trait A can be observed. These are the explanations typically investigated in evolutionary research. Let these be ***Type I*** explanations. The second possibility is that an amount of variation could in principle exist, but it so happens that it has never come about (related to the idea of evolutionary lag (Smith, 1978) but on longer time scales). Since natural selection never acted on trait B, we observe only trait A. Let these be ***Type II*** explanations. Third, variation in a trait could be impossible because of biophysical or biochemical limitations. In this case no alternative to trait A could possibly exist—there is a constraint (Smith, 1978; Gould, 1980). Let these be ***Type III*** explanations. (Plasticity can also be considered a trait in this framework.)

The original evolutionary theories of aging are Type I explanations. They start from a (hypothetical) non-aging phenotype and argue how the aging phenotype could invade (Medawar, 1952; Williams, 1957; Hamilton, 1966; Kirkwood, 1977), as in Kirkwood’s *mobbit* (Kirkwood, 1999). This implies that human-like complex organisms might evolve without aging if only a limited number of genes were different (mutation accumulation and antagonistic pleiotropy), or if the available energy were allocated differently (the disposable soma theory). One of the main purposes of the present paper is to argue the alternative: *there are some complex organisms such as humans for which no variation could possibly exist such that they do not age.* This is the evolution of unmaintainability, a Type III explanation.

## Systemic Constraints on Physiology and Evolution

Genes, biochemistry, and physiology act in a world governed by the laws of physics and chemistry (Thompson, 2014). The product of a gene, whether that be a protein, microRNA, or any other substance, not only has to obey these laws, but also has to fit in the overall cellular metabolism, which may severely restrict DNA action (de Lorenzo, 2014; de Lorenzo, 2015). Likewise, there are no genes that invert gravity. If the laws of physics and chemistry do not change over time, which seems a reasonable assumption, then they are the same for parent and offspring. If these laws leave their traces in organismal physiology, then not all similarities between parent and offspring are heritable. It is such constraints that give rise to Type III evolutionary explanations.

Beyond the constraints of chemistry and physics, there exists a series of more specific biological constraints, most notably phylogenetic inertia, interoperability, and developmental constraints.

### Phylogenetic Inertia

Genomes do not evolve *de novo*, but rather through small modifications to existing genomes. Adaptation must happen through continuous change in which all intermediate forms are viable in their current environment. Accordingly, the history of a lineage as reflected in its genome may impose major constraints on the phenotypic space that is accessible through natural selection (Blomberg and Garland, 2002). For example, no mammals have the remarkable regenerative capacities that starfish do. This likely reflects a moment in the history of the mammalian lineage at which the flexibility to evolve regeneration was sacrificed for other properties of development; while it might be theoretically possible to imagine a series of genetic changes that would restore extreme regeneration, this will not happen in nature.

### Interoperability

An organism is finely tuned, integrating countless molecules affecting numerous systems that together coordinate homeostasis at levels ranging from sub-cellular to organismal. By necessity, these systems interact, meaning that changes to one system can affect others. This presents a major constraint on the ability to optimize components piecemeal. For example, the inflammatory cytokine interleukin-6 plays important roles in acute inflammatory response to an infection (Helfgott et al., 1989), in the senescence-associated secretory phenotype of senescent cells (Laberge et al., 2012), and in the chronic low-grade inflammation (“inflamm-aging”) that plays a role in many chronic diseases (Cohen et al., 2018). Its implication in pathways is different in each of these roles, and so any mutation that affected one role would also be under selection for its impacts on the other roles. Interoperability manifests itself hierarchically (cells need to interoperate with tissues and molecules, for example), across similar levels of organization (for example between organs), and environmentally (organisms need phenotypes that function under different conditions). Accordingly, the interoperability constraint affects nearly everything, often in ways that are very specific to a given species’ biology and ecology.

### Developmental Constraints

Adult phenotypes must be arrived at through a series of steps during development. Only phenotypes that can be arrived at by tweaking the developmental process are feasible. Phylogenetic inertia and interoperability constraints pertain also to the process of development, linking these constraints.

These three types of constraints can be quite forbidding, so much so that they might appear to give rise to Type III evolutionary explanations. They do however test the difference between Type II and Type III explanations, as we could imagine that in a much different evolutionary history, constraints could have been different. In any defined physiological and genetic setting however, these are hard, Type III constraints. For example, arthropod size is constrained by the ability to deliver oxygen to tissues as surface area-to-volume ratio decreases; this constraint was circumvented in tetrapods by the evolution of the lung. But within any given arthropod species, the size constraint can be considered Type III for all practical purposes.

When do systemic constraints arise? The design and construction of a complex organism versus products designed by humans are fundamentally distinct. In the latter case, there is a blueprint to which the engineer can refer at any moment, and the detailed instructions which the engineer tries to follow as closely as possible. In the former case, the information consists in procedural instructions without reference to a greater scheme of things: “There is (…) information in a fertilized egg (in genes, in molecular structures, and in spatial variations in the concentrations of particular chemicals), but this has no simple relationship with how the final built body will look. (Rather) the information has the effect of controlling the sequence of events that will follow.” (Davies, 2014).

The cues that lead to developmental phenomena are provided by developing tissue, against the background of the environment the embryo finds itself in: for mammals the mother’s womb. This means that repair capacity, at least in as much as it relies on *re-making* components, critically depends on the presence of conditions that may no longer exist in an adult organism. Informational cues, physical space, and accommodating complexities that were instrumental in the formation of a tissue may no longer be present in an adult organism. It is not without reason that the switching on and off of genes during development is so carefully timed: the moment these genes are active may be the only moment that expression has the desired effect (Davies, 2014).

Just being an adult changes things, too. Take the formation of the brain. A newborn human hardly relies on its higher brain functions for survival. Higher brain functions develop because brain cells are created in overabundance and make connections in overabundance. Nature then tests these connections and confirms or discards them during synaptic pruning. Those connections (and cells) that are useful are retained and reinforced, while the other connections are removed (Davies, 2014). In an adult organism, the circuits formed during pruning have become vital for its survival and cannot be repaired by repeating the same procedure; the organism would be helpless prey. For these and other reasons, the result of development is an organism with a physiology that restricts repair. Complex organisms such as humans have laid layer upon layer of complexity, inhibiting repair and leading to the loss of simple regenerative capacity of tissues.

## Unmaintainability as an Emergent Property of the Complexity of Life

Even if we remain, for the moment, within the realm of aging caused by insufficient repair of damage in the classical, three-dimensional sense, after development repair may be severely limited by the wholesale physiological organization of an organism, due not only to the already mentioned constraints on resources, but also to the following.

**Information**. Any repair process requires guidance as to the desired state of the organism (Kirkwood, 1981). If repair amounts to replacement of a damaged molecule, then it is clear where that information could come from: new molecules are synthesized using the pathway involved in regular synthesis, while old ones are discarded. But it is not always that straightforward. In insects, the mother copies spatial information about her own body into the eggs as gradients in concentrations of molecules [(Davies, 2014), notice the non-DNA inheritance]. The Bauplan of the offspring is derived from these gradients. If the body of a mature insect is sufficiently damaged, the information on the desired state is irretrievable: repair is limited.

**Diminishing Marginal Returns**. If a dirty floor is swept, the first pass with the broom will get up most but not all of the dirt; the second pass will get most of what is remaining, but much less than the first pass, and so forth. At a certain point, one decides to stop sweeping because the amount of additional dirt swept up is not worth the effort. This principle of diminishing marginal returns applies equally well to many repair and maintenance processes. For example, the energetic costs of repair become relatively higher the rarer damage types are (requiring more brooms and more sweeps), and thus the greater the investment required (Gems and McElwee, 2005). Diminishing marginal returns can also interact with other constraints, such as damage detection below.

**Damage Detection**. In addition to “knowing” the desired state, the repair machinery should also be able to detect a deviation from the desired state. (We are grateful to Diana van Heemst for pointing this out.) This could be by comparison to a blueprint, the sensing of a disturbed function, or through the local release of cytokines. Often the detection mechanism will only indicate that damage exists, without conveying its exact nature, limiting repair.

**A Compatible Physical and Chemical Environment**. A repair process needs to access the site of injury and to operate there, which may be limited by the function of the tissue to be repaired. Humans have to counter gravity and other forces, and structures that handle those forces (bones, tendons, actin filaments) cannot be (re)moved, or can be (re)moved only to a very limited extent. Similarly, repair machinery that deals with arterial damage should be able to operate in the sheer stress caused by the blood flow. Likewise, any repair machinery may need a specific chemical environment that interferes with the normal functioning of the damaged tissue. Repair in an artery takes place in an overall environment with a pH of 7.41; altering this pH would interfere with the blood’s function of carrying oxygen, as oxygen dissociates from hemoglobin in an acidic environment (Bern et al., 2004). Furthermore, sending a cell into apoptosis and the communication of the apoptotic cell with its surroundings may interfere with normal cellular communications (Suzanne and Steller, 2013).

**The evolution of the repair process**. Even if a repair process could exist uninhibited by the constraints above, how would it come about?

Evolution works with the variation available as Gould proposed with the concept of exaptation (Gould and Vrba, 1982), and as Jacob expressed in his concept of “evolutionary tinkering” (Jacob, 1977). An example of tinkering is found in the brain, which consists of neurons that shape the electric connectivity of the brain, and supporting cells that insulate and feed the neurons, or have an immune function. Microglia are the supporting cells that perform the immune function, but their role encompasses much more than immunity (Marin and Kipnis, 2013). They secrete tumor necrosis factor alpha (TNF-α) into the synapse, where neurons communicate, which affects neuronal potentiation (Jebelli et al., 2015) and neuron network stability (Stellwagen and Malenka, 2006). Thus, it seems that a cell whose primary, historical function was immunity uses a molecular substance, TNF-α, whose primary, historical original function was immune regulation, to improve the function of the brain. Microglia (and TNF-α) were present in the brain because of their immune function, but because TNF-α happened to affect neuronal activity, a novel function evolved. This scenario is unconfirmed but arguably more likely than the emergence, through pure random variation, of some hypothetical cell type that similarly adjusts neuronal activity (Gladyshev, 2016). A comparable principle likely applies to many repair processes: they may have been adapted from other functions rather than being tailored to a specific type of damage. Accordingly, repair is unlikely to be perfect.

Straightforward exchange of components may be feasible in some organisms, but not others (Schaible et al., 2014), and not all types of repair consist in routine replacement. For instance, the arterial fatty streak or the extra-cellular plaques in Alzheimer’s disease have no physiological function, but are (by-) products that should not be there in the first place. They should be removed rather than replaced, which requires a separate, new repair mechanism, or a complete reconstruction of an entire new organ. It is unclear how this could be achieved mechanistically, and even less clear which evolutionary-historical path could lead to such invention.

## Moving Beyond Damage

Going beyond the boundaries of damage being the cause of aging complicates matters further still. It is now well understood that life is a complex system in the formal sense: composed of multiple interacting elements with feedback loops, a hierarchical structure, non-linear dynamics, and emergent properties (Kitano, 2002). Emergent properties are properties of a system that are not evident by considering lower-level components individually or additively. For example, blood pressure is an emergent property of the circulatory system that cannot be understood as a simple property of cells in the vasculature. Consideration of an organism as a complex system can have a radical impact on the questions we ask: it is not sufficient to decompose an organism into constituent pieces, without asking how the pieces all interact as an ensemble to generate function.

Joining a complex systems perspective on biology with the above considerations on constraints and unmaintainability, a new hypothesis emerges for how unmaintainability (and thus aging) could evolve. A key property of many complex systems is robustness (Kitano, 2007; Kriete, 2013), defined here as the capacity to maintain stability of key aspects of a system in the face of perturbations and challenges, and thus an approximate converse to fragility. This is particularly crucial for biological systems, which, in order to survive and reproduce, must maintain dynamic equilibrium in the face of ever-changing conditions. Low robustness is not necessarily concomitant with unmaintainability or aging—for example, a lack of robustness to a heat shock or to starvation could result in abrupt death, regardless of age. Nonetheless, most biological networks are highly redundant and buffered (Nijhout et al., 2017), explaining why so few genes are lethal when knocked out, and may not even produce noticeable effects under normal conditions. In this context, when the tolerance of the system is exceeded (i.e., insufficient robustness), the consequence is likely not death, but an adverse change to network state. This can manifest either as a shift to an alternative state that is less functional or less robust itself, or as a cascading series of failures that are not immediately fatal but set the organism on a downward spiral. For example, the vertebrate corticosteroid-based stress response creates a cascade of changes (blood pressure, kidney function, etc.) that are generally adaptive short-term, but when the tolerance of the system is exceeded by a chronic stress, these same changes impact many known aging mechanisms (telomeres, inflammation, oxidative stress, etc.) (McEwen, 1998; Zalli et al., 2014; Mocayar Marón et al., 2019; Sterling, 2020).

Theoretical work on robustness in complex systems, particularly in highly optimized tolerance systems such as living organisms, has shown that robustness is usually achieved with certain trade-offs: robustness to one type of perturbation may come at the cost of increased susceptibility to another, or may be achieved with increased resource investment, or a decrease in functionality, etc. (Zhou et al., 2005; Kriete, 2013). As evolution explores the space of robust potential phenotypes, constraints such as those outlined above pose further limits on the portions of phenotypic space that are accessible, and thus impose additional limits on maintainability. The robustness of the regulatory networks that maintain dynamic equilibrium within organisms over time is thus expected to be imperfect (Cohen, 2016). When a sufficient number of vulnerabilities exist in the system, it becomes unmaintainable, and aging results.

On the surface, this explanation may seem to agree with classic resource allocation and trade-off theory (and indeed it is complementary), but there are several important distinctions. First, classical trade-off theory would seem to imply that, with sufficient resource investment, or with different genes, aging might be avoided altogether. This is not predicted here: the nature of robustness is to be imperfect, and resource allocation is only sometimes expected to be the important factor with which robustness trades off. Infinite resources would not likely stave off aging much under this hypothesis, nor would a different set of genes. A thought experiment illustrates this. Imagine Earth is colonized by a dominant alien species that attempts to breed humans to become immortal. Only lineages stemming from the longest-lived individuals are allowed to persist, thereby weakening the selection shadow on the current human lifescale and largely counteracting mutation accumulation. Classical theory posits no force limiting evolution toward immortality, whereas the Danaid theory predicts that human lifespan would evolve asymptotically toward a limit defined by unmaintainability and systemic constraints: with the existing physiological *Bauplan*, aging is not avoidable.

Second, aging need not depend on any specific mechanism or pathway, or even any clearly demarcated set of pathways. It is the system that is robust (or not), the system that dysregulates (or not). Aging could emerge even in the absence of clear-cut mechanisms (though mechanisms are not precluded). Third, classical trade-off theory predicts a single fast-slow continuum of life histories across the tree of life, which is clearly not observed. A complex systems perspective on robustness implies that the specific physiology of each taxon will determine the landscape and strength of the trade-offs involved. Hence, some taxa [e.g. mammals (Gaillard et al., 2016)] would display universal aging, a fast-slow continuum, and clear trade-offs, whereas others might show very different patterns, ranging from apparent lack of aging (e.g., some sharks, some turtles) to taxonomically fine-scale variation in the presence of aging and the strength of trade-offs (e.g. ray-finned fish).

The theory we propose portrays aging as an arc or trajectory. Similar trajectories are known in development/morphogenesis (Davies, 2014), and are increasingly recognized in the immune system (Franceschi et al., 2017; Fulop et al., 2017) and in neurobiological and cognitive trajectories (Suzanne and Steller, 2013). We propose that such arcs are present in a wide array of aspects of organismal biology and physiology. Homeostatic networks may change with age (Dansereau et al., 2019), as may the functions of various organs, not because they are degrading or failing but because the changes are the natural consequence of the arc of that system, and of its integration with all the other systems following their arcs. The idea that all aspects of aging are deleterious may reflect a cultural bias (Cohen et al., 2020c), and many aspects may indeed be adaptive (Le Couteur and Simpson, 2011). For example, mammalian sarcopenia is widely seen as a major aging-related pathology, but it can also be conceived of as a mechanism to reduce energy requirements, and thus potentially adaptive in the broader context of the arc of the organism, despite its adverse consequences on muscle function. The Bauplan of an organism is the unit on which the arc takes its course, and unmaintainability is an emergent property of the multiple trajectories evolving concomitantly within that Bauplan; this may explain why aging is so different in organisms without a fixed Bauplan.

We thus argue that the aging process can be linked to the complexity of life: that it can be an emergent property of life, with unmaintainability generated by the numerous competing demands on the organism and/or its regulatory complexity across the arc of development and adulthood. This does not mean that all complex organisms will necessarily age, nor that all systemic constraints require complexity, but rather that complexity can in some cases be a driving force for aging at the interface of evolutionary and mechanistic levels. Additonally, complexity is not the only potential reason aging evolves, nor the only potential mechanism: clearly some simpler forms of life (e.g. yeast, *Caenhorabditis elegans*) do age, whereas some more complex forms (e.g. some vertebrates) do not. It is time to move beyond the attempt for silver-bullet explanations of aging. Multiple forces interact, with different strengths in different taxa, to generate patterns of aging.

At first glance, it might appear that the Danaid theory (Table 1) is simply a special case of the declining force of selection. At some point, organisms evolved greater complexity or other traits that made them less maintainable/more susceptible to aging; natural selection nevertheless favored these more-aging-susceptible variants, making it appear that the declining force of selection must always be upstream. However, this ignores two key points. First, it is not necessarily the case that complexity will shorten lifespan. It is fully possible that aspects of complexity could evolve and become fixed in a lineage under conditions where they have no impact on lifespan, or even improve lifespan (Wensink et al., 2014b), and that any lifespan-limiting impacts could become apparent only in daughter lineages with specific environmental or physiological conditions. The evolutionary origins of the complexity/unmaintainabilty are thus not crucial to consider. Second, as noted, we are not solely concerned with explaining why aging first evolved, but also why it differs as it does across taxa. Regardless of how complexity and unmaintainability were originally selected for, we argue that once present they have continuing impacts on how aging evolves subsequently (including the way it may disappear or arise anew in a lineage), and are thus germane to an evolutionary theory of aging but distinct from the declining force of selection. For example, it would appear that some organisms of moderate to high complexity do not age, at least within observable/relevant timescales (minimally hydra, but likely many vertebrates too (Congdon et al., 2003; Jones et al., 2014; Sauer et al., 2021)). How might that complexity affect the subsequent evolution of aging and lifespan in their descendant lineages?

## The Comparative Biology and Evolution of Unmaintainability

Classical theories rely on the selection shadow to explain aging. With some latitude for how the different sub-theories might interact, the prediction is thus that selection shadow should correlate tightly with aging rate. While this may indeed be the case within certain clades (mammals or mammalian orders, for example), it is almost certainly not the case across wider swaths of taxa.

Information on the comparative biology of aging comes from two types of sources: demographic data across a broad array of species (Jones et al., 2014; Salguero‐Gómez et al., 2014; Salguero-Gómez et al., 2016; Scheuerlein et al., 2017), and experimental/lab work on mechanisms in diverse species. The former has the advantage of covering many more species and more branches of the tree of life, but the disadvantage of an inability to disentangle physiological aging mechanisms from other mechanisms that cause aging-like signatures in demography. Negative senescence due to factors such as size might partially cancel out mortality increases or fecundity decreases due to physiological aging, but, if aging is inexorable and leads to inevitable death, at some point the physiological aging should become demographically apparent if observation time suffices.

The picture painted by demographic studies is as follows: Aging appears to be present in all birds and all mammals. Potential exceptions such as naked mole-rats (Ruby et al., 2018) may not have been followed long enough; we remain agnostic. There are some taxa that appear not to age, or where lack of aging is widespread, such as sharks, crocodilians, and turtles (Finch, 1994; Jones et al., 2014). In many taxa, aging is more of a patchwork: in ray-finned fishes and plants, there are clear examples of species that appear not to age, and clear examples of species that age very quickly, often without a clear taxonomic pattern, though data are not yet complete enough to thoroughly evaluate phylogenetic signal. There are many examples of unusual aging patterns or lack of aging in invertebrates and plants with clonal reproduction (Shefferson et al., 2017); mechanistic studies generally do not contradict these findings, and provide better resolution for the physiology. Note, for example, that plants can handle polyploidy and aneuploidy, and even use it to their advantage (Tudge, 2006), while mammals cannot. Within mammals, exceptional lifespans of naked mole-rats, blind mole-rats, and bowhead whales appear related to specific adaptations, unique in each species, to avoid certain aging mechanisms (Gorbunova et al., 2012; Takasugi et al., 2020; Cooper and Gorbunova, 2021). It is unclear whether the adaptations discovered represent ways to constrain the most-limiting aging mechanisms in each species, or are a small subset of a large number of adaptations to limit a large number of mechanisms. Current evidence suggests, but does not definitively prove, that these adaptations can succeed in slowing but not stopping aging in mammals. Broadly, for both mammals and birds, correlations between lifespan and body size/metabolic rate/reproductive rate do suggest some role for trade-offs in structuring lifespan, though the associations are weak enough for other factors to play important roles.

Indeed, there appear to be a variety of factors that explain unusual aging patterns in different species, rather than a single unifying explanation. The exceptional lifespan of the ocean quahog, *Arctica islandica*, (record: 507 years) seems to be the extreme of a continuum. Various clam species with different lifespans differ continuously in their ability to avoid protein aggregation and loss of proteostasis (Ridgway et al., 2011; Ungvari et al., 2011; Treaster et al., 2015). The lack of aging in hydra appears to be dependent on both species and environment (Martínez, 1998; Martínez and Bridge, 2012). In killifish, as noted above, short lifespan appears to evolve repeatedly but through different mechanisms: breakdown in housekeeping genes across the genome as selection relaxes on the ability to maintain physiology for more than several months (Cui et al., 2019). In taxa such as lepidoptera and bees, wear-and-tear on wings may be a major factor limiting lifespan (Rueppell, 2009), and is probably not closely related to the conserved genetic pathways that appear limiting in yeast, fruit flies, nematodes, and mammals.

This portrait of comparative aging suggests that there is no single explanation that will concisely summarize everything. There are conserved genetic pathways, and these do modulate aging in some species, but not all. In some taxa, trade-offs have a large role, in others a weaker role. There are few if any universal mechanisms, but in at least some species controlling the key known mechanisms is part of the reason they can evolve exceptional lifespans. Some constraints, while by definition inevitable, may nonetheless be modulated. For example, it is likely impossible to eliminate DNA damage completely, but it can be drastically reduced with appropriate investments in protection and repair. (By contrast, cellular senescence is a programmed pathway, and could presumably be eliminated completely by one or several key mutations. Cellular senescence is thus best thought of as an adaptation, for example to reduce cancer risk and for pruning in brain development.) Both adaptations and modulable constraints may be subject to trade-offs.

In this context, we argue that unmaintainability is the missing piece of the puzzle that allows us to make at least a little sense of this patchwork—for example, why mammals show a slow-fast life history continuum (Gaillard et al., 2016), but sharks do not. For any given taxon, the fundamental question it faces in the evolution of lifespan is, what are the constraints, i.e., what factors will limit lifespan regardless of selection and physiological adaptation? The answer to this question will be fundamentally different for a hydra, a butterfly, a fruit fly, a pine tree, a shark, and a mammal.

Above, we have given examples of the types of constraints that may apply, both generally and in regards to aging. One that deserves particular attention is the type of constraint arising from the structure of the homeostatic networks specific to each taxon. For example, in mammals, high levels of glucose are toxic and generate advanced glycosylation end products, which appear to contribute to aging; in birds, similar glucose levels are tolerated, and indeed necessary to sustain the metabolism associated with flight (Holmes et al., 2001). Thus, despite numerous similarities between bird and mammalian metabolic networks, there are also fundamental differences that generate regulatory weak points for each taxon. Metabolic syndrome and inflamm-aging, for example, appear to be mammalian phenomena. In other words, the regulatory networks that have evolved in each taxon have unique strengths and weaknesses; in some cases, these weaknesses will create ways the system is unmaintainable, and thus represent constraints on the evolution of lifespan.

Accordingly, the model we propose is that each taxon has a unique set of unmodulable constraints, modulable constraints, and adaptations that may affect aging. The constraints may often be related to the structure of regulatory networks and how this interacts with the taxon’s ecology/niche (e.g., the need of a bird to migrate, insectivorous diet, etc.). Within the broad framework set by the constraints, trade-offs and drift/stochasticity then operate to produce the specific aging patterns of each species. Note that we discuss taxon-specific constraints rather than species-specific constraints because in some cases the relevant variation is mostly at higher levels. For example, it would appear that there is relatively little variation in the constraints within mammals, or within birds (the basic physiological and biochemical architecture being relatively similar). In contrast, the constraints may be different from one hydra species to another, though it is hard to say for certain (Martínez and Bridge, 2012).

The constraints arising in a given taxon may have their origins in relatively random factors early in the taxon’s evolution, which have subsequently become fixed. For example, early mammals had a much more limited set of ecological niches for a long time and were presumably relatively short-lived; it would be unsurprising that their physiological architecture evolved containing constraints that imposed important limits on the subsequent evolution of lifespan as the taxon diversified. Differences in constraints across taxa create a patchwork of how unmaintainable each taxon is. Within this broader framework, trade-offs and other forces can operate to adjust aging. In some sense, this is a rather unsatisfying answer and explanation: the explicit prediction is that local, hard-to-predict and hard-to-identify factors are responsible for the diversity of aging patterns across the tree of life, and thus there is no broad theory that will explain the evolution of aging more generally. On the other hand, ours is a coherent explanation for why a simpler theory should probably never have been expected to be sufficient, and is consistent with the vast bulk of what is known about aging at many levels: genetic, molecular, physiological, demographic, and evolutionary.

## Summary and the Next Steps

In Greek mythology, the Danaids were the 50 daughters of Danaus. They were supposed to marry the 50 sons of Aegyptus, but all but one of them killed their husbands on their wedding night. For this, they were condemned to spend eternity carrying water in a perforated vessel or sieve, and became a metaphor for futility. We call our theory the “Danaid theory of aging” because we propose that organismal biology and physiology are like the leaky vessels of the Danaids, unable to hold life in them eternally due to constraints in their basic structure. This metaphor creates a clear contrast with programmed theories of aging (which would imply that the Danaids would pour water out of the vessels intentionally for some reason), and with classical evolutionary theories relying on trade-offs and selection pressure (which might imply that the Danaids simply did not pay enough attention to avoid sloshing and spilling the water, or that they chose to accept some spillage because they wanted to run while carrying the water and accepted the consequences). We also add a nuance to the metaphor: some vessels (organismal physiologies) are leakier than others (and a few might even be completely unperforated), making them quite variable in their ability to hold water (life).

In summary, the Danaid theory of aging proposes that, in many taxa, aging is an inevitable consequence of how life has been constructed in that taxon, for reasons (constraints) largely independent of selection on aging and lifespan. Aging and lifespan are then further modified within each taxon by optimization and mutation accumulation as predicted based on the declining force of selection with age. The underlying constraints are expected to vary across taxa in ways that may appear arbitrary, reflecting developmental and regulatory requirements as well as the vagaries of chance across the ancestral lineage.

### A famous quotation goes

“It is indeed remarkable that after a seemingly miraculous feat of morphogenesis a complex metazoan should be unable to perform the much simpler task of merely maintaining what is already formed.” (Williams, 1957).

Often still seen as the core question that an evolutionary theory of aging should seek to answer, this misses the point. Why would maintenance be “much simpler” and “mere”? Cues present during morphogenesis may no longer be there. Development may have closed repair options. And complex regulatory networks may simply slide out of equilibrium through repetitive perturbations, without a clear way of putting them back. Hence, the quote might just as well read:

“Clearly, after the miraculous feat of morphogenesis many complex metazoans are unable to perform the virtually impossible task of maintaining what was formed.”

But even more, we noted in Section *Systemic Constraints on Physiology and Evolution* that evolution can only pick up options that it stumbles across (Jacob, 1977). The process of morphogenesis already exists in any organism for historical reasons (Turke, 2013): Whether organisms age or not, they die. Hence, whether or not chronologically old organisms are also biologically old, the necessity to reproduce exists. It is not at all impossible, therefore, that in the light of the restrictions on repair discussed here, nature patches for the short term, while in the long term it applies one all-encompassing repair mechanism: the creation of a new organism. Hence, an even more apt variation on the quote above could read:

“Rather than performing the virtually impossible task of maintaining itself, the complex metazoan repeats the miraculous but proven feat of morphogenesis.”

We are not the first to mention the role of constraints in shaping the evolution of aging. The hyperfunction theory does so more specifically (Blagosklonny, 2012; Gems and de la Guardia, 2013), and trade-offs themselves are a form of constraint (Cohen et al., 2019). However, we feel that the broad picture that persists in the literature is that constraints are an afterthought, a detail that can be ignored, or worse: invoked whenever standard explanations fail. We argue that thinking carefully about what constraints are present can fundamentally alter how we understand the evolution of the aging process, and that such efforts have rarely been undertaken.

As we have emphasized, the exact mechanisms of aging, and hence its evolution, are expected to vary greatly from taxon to taxon, which complicates the task of making predictions. But across the tree of life, reproduction remains the ultimate repair. Some organisms also seem capable of keeping their own body in perfect shape, while others do not. In finding a new theory of the evolution of aging the central question thus becomes:

“Why is it that some organisms (species) seem incapable of doing inside their body what they are perfectly capable of doing outside their body: to create a perfectly healthy organism?”

To answer that question, we need to map the diversity of mechanisms and pathways across the tree of life. Such efforts are beginning, including in this Research Topic, but the task is daunting because of the variety of potential mechanisms and the large number that may be highly specific. Efforts to study mechanisms in unusual species (ocean quahogs (Ungvari et al., 2011), naked mole-rats (Tian et al., 2013), etc.) have already proven highly fruitful; the next challenge is to identify unusual mechanisms and quantify their importance. This could lead to eventual empirical tests of this theory, and to further theory building. If unusual or non-canonical aging mechanisms are rarely if ever important for a species’ demography, they may be negligible, in contradiction to our theory. If the Hallmarks of aging are shown to be not just present in most/all species, but sufficient explanations, and modulable, our theory would be disproven. Conversely, our theory will be supported if increasing data show variation in aging mechanisms/constraints in different taxa (Omotoso et al., 2021), often at variance with the forces of selection, and if we continue to uncover a larger and larger role for the breakdown in homeostatic mechanisms or other basic constraints in shaping the aging process. It is time for theory on the evolution of aging to incorporate what is known about development, physiology, genetics, and comparative biology, and to acknowledge explicitly that constraints could result in aging even in the absence of the selection shadow, and thus even in the absence of the classical theories.
